# Evaluation of early arterial wall lesions by elastography parameters in spontaneously hypertensive rats

**DOI:** 10.1186/s12872-023-03135-9

**Published:** 2023-03-08

**Authors:** Jinping Liu, Lanyan Qiu, Yuan Su, Hong Zhang, Xianquan Shi, Xiangdong Hu, Linxue Qian

**Affiliations:** Department of Ultrasound, Beijing Friendship Hospital, Capital Medical University, No. 95 Yong An Road, Beijing, 100050 China

**Keywords:** Hypertensive rats, Early, Arterial lesions, Ultrasound, Elastography parameters

## Abstract

**Background:**

Arterialsclerosis caused by hypertension can lead to many complications, such as heart attack, stroke and so on. Early diagnosis and treatment of arterialsclerosis can prevent cardiovascular and cerebrovascular diseases and improve the prognosis. The present study aimed to explore the value of ultrasonography in evaluating the early lesion of the local arterial wall in hypertensive rats and identify useful elastography parameters.

**Methods:**

A total of 24 spontaneously hypertensive rats (SHR), 10-, 20-, 30-, and 40-weeks-old, were used in this study, with 6 rats in each group. Blood pressure was recorded using the Animal Noninvasive Blood Pressure Measurement System (Kent company, model CODA, USA), and the local elasticity of the abdominal aorta of rats was measured using a ultrasound diagnostic instrument (VINNO, Suzhou city, China). According to the histopathological results, SHR were divided into two groups: the normal arterial elasticity and the early arterial wall lesions. Mann–Whitney U test was used to compare the differences in elastic parameters and influencing factors between the above two groups, and receiver operating characteristic curve (ROC) was used to analyze and judge the value of each elastic parameter in evaluating early arterial lesions.

**Results:**

A total of 22 cases were divided into two groups: 14 in the normal arterial elasticity and 8 in the early arterial wall lesions. The differences in age, blood pressure, pulse wave velocity (PWV), compliance coefficient (CC), distensibility coefficient (DC), and elasticity parameter (EP) between the two groups were compared. The differences in PWV, CC, DC and EP were statistically significant. Subsequently, the ROC curve analysis was performed for the above four evaluation indexes of arterial elasticity; the results were as follows: the area under the curve of PWV, CC, DC, and EP was 0.946, 0.781, 0.946, and 0.911, respectively.

**Conclusions:**

Early arterial wall lesions can be evaluated by ultrasound measurement of local PWV. PWV and DC can accurately evaluate the early arterial wall lesions in SHR, and the combined application of the two can improve the sensitivity and specificity of the approach.

## Background

The incidence of hypertension has increased in recent years, and high blood pressure is the primary factor leading to ischemic heart disease and premature death. High blood pressure also leads to heart failure, stroke, atrial fibrillation, chronic kidney disease, and peripheral vascular disease and is a major risk factor for cognitive decline [1]. Arteriosclerosis is a complication of hypertension. Increased arterial stiffness is an independent risk factor for organ damage and cardiovascular disease [2] and is closely related to stroke and coronary heart disease [3]. Based on the significance of early diagnosis and prevention of atherosclerosis, the study of arterial elasticity function has become a research hotspot [4–6].

The early manifestations of atherosclerosis include decreased arterial wall elasticity, hyperplasia of collagen fibers, and formation of endodermal lipid vacuoles [2]. The indices for evaluating vascular elasticity include pulse wave velocity (PWV), compliance coefficient (CC), distensibility coefficient (DC), elasticity parameter (EP), and pulse pressure (PP). Presently, the most commonly used index of arterial elasticity is PWV. Due to its advantages, such as safety, extensive use, and non-invasiveness, PWV is considered a critical indicator and the gold standard that can accurately reflect arterial elasticity [7–10]. However, the measurement method is based on the two-point approach (regional PWV), i.e., the average conduction velocity of a certain segment of the blood vessel. This method assumed that the direction of the blood vessel between the two measurement points is straight, and the hardness of each point on the path is uniform. Since vascular hardness is not uniform, the local hardness can only reflect the local mechanical characteristics of the vessel. Thus, in recent years, the importance of local PWV has been recognized as a measurable physiological parameter. The local PWV measurement is based on a short segment of an artery and represents an early diagnosis tool that can identify the local stiffness of the arterial wall [11]. Local PWV has emerged as a robust independent predictor of all-cause and cardiovascular mortalities [12]. Hitherto, some ultrasonic devices have been utilized to measure the local pulse wave velocity [13–15].

Although PWV has been shown to evaluate arterial elasticity in hypertensive patients, only some clinical studies have been conducted in human populations [16–19]. The present study is an animal experiment; the innovation of this study is that local PWV was used to evaluate arterial elasticity with histopathological gold standard control, and multiple elastic parameters could be obtained simultaneously. Herein, the elasticity of local abdominal aorta in spontaneously hypertensive rats (SHR) was evaluated using a ultrasound diagnostic instrument, and multiple elasticity parameters, including PWV, CC, DC, and EP were obtained. The combined application can improve the sensitivity and specificity of diagnosis. In addition, the method used in this study was simple and easy to operate. The repeatability test was carried out in the early stage, and the results of different operators were consistent [20]. Thus, this method could be used to measure arterial elasticity in hypertension patients in the future.

## Methods

### Animals

According to the international, national, and institutional rules, the study was performed considering animal experiments, clinical studies, and biodiversity. The study protocol was approved by the Ethics Committee of Beijing Friendship Hospital, Capital Medical University, Beijing, China.

A total of 24 spontaneously hypertensive rats (SHR) (including 12 males and 12 females) were purchased from Beijing Huafucang Biotechnology Co, Ltd. The rats were fed under a specific pathogen-free (SPF) grade environment in the Animal Experimental Center of Beijing Friendship Hospital, Capital Medical University. SHR rats were divided into four groups: 10-, 20-, 30-, and 40-weeks-old, with six animals in each group, according to the rule that blood pressure increases with increasing age.

### Determination of caudal artery blood pressure

The rats were preheated at 40 ℃ for 15 min and the systolic blood pressure (SP) and diastolic blood pressure (DP) of the caudal artery was measured by the American Kent Company Animal Noninvasive Blood Pressure Measurement System (model CODA) at 10, 20, 30, and 40 weeks after resting and wakefulness [21]. Each animal was assessed five times consecutively at 1–2-min intervals.

### Measurement of elasticity of rat abdominal aorta by ultrasound

**Fig. 1:**
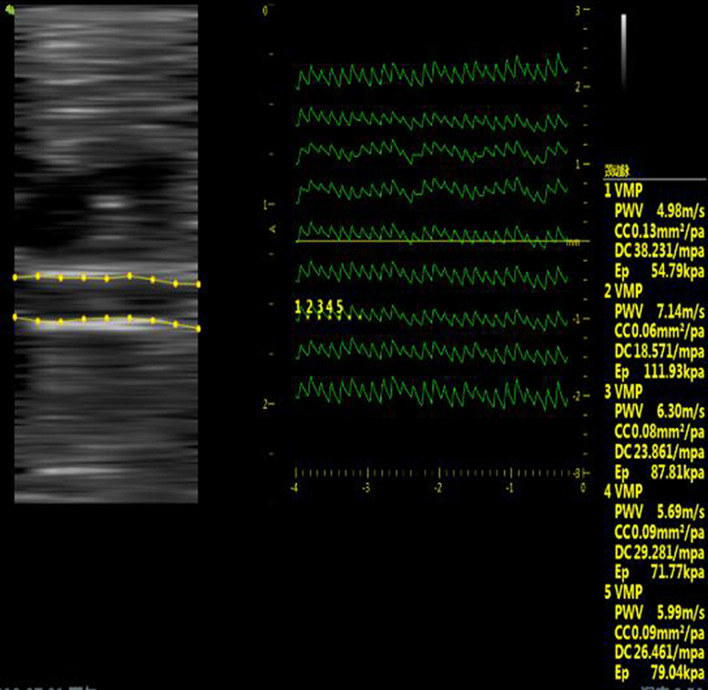
Ultrasound evaluation of elasticity of rat abdominal aorta (The left panel shows a B-mode image of the scan line, and the right panel shows the pulse of the vessel wall at the position of the scan line in real time Wave spectrum, get at least one cardiac cycle of the pulse wave after freezing)

The elasticity of the abdominal aorta in rats was measured using a VINNO M80 ultrasonic diagnostic instrument with probe X6-16L (frequency 6.5–18 MHz). The main abdominal aorta was identified in the left abdomen of the rats under anesthesia. The abdominal aorta was maintained parallel to the probe to display the long axis of the abdominal aorta. After the position was determined, the “PWV” key was started. The movement track of the abdominal aortic wall on each sampling line was presented on the image in the form of an expansion wave, showing the expansion wave of the movement track of the abdominal aortic wall in at least five stable cardiac cycles. The measuring cursor was used to indicate the position of “trough to trough” in one cardiac cycle. The local PWV, CC, DC, and EP of the vessel wall were measured five times for each measurement. Then, the maximum and minimum values were removed, and the average value of the remaining three times was calculated (Fig. 1).

### Histopathological detection of the abdominal aorta in rats

**Fig. 2:**
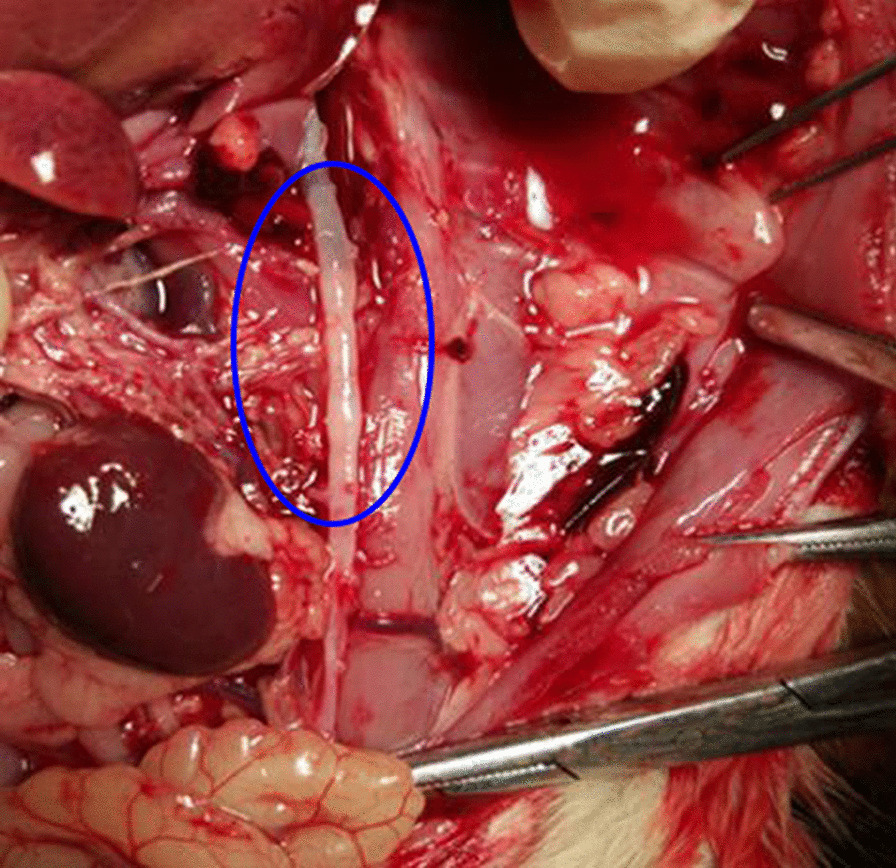
Anatomical view of abdominal aorta in spontaneously hypertensive rat (inside the blue circle is the abdominal aorta)

**Fig. 3:**
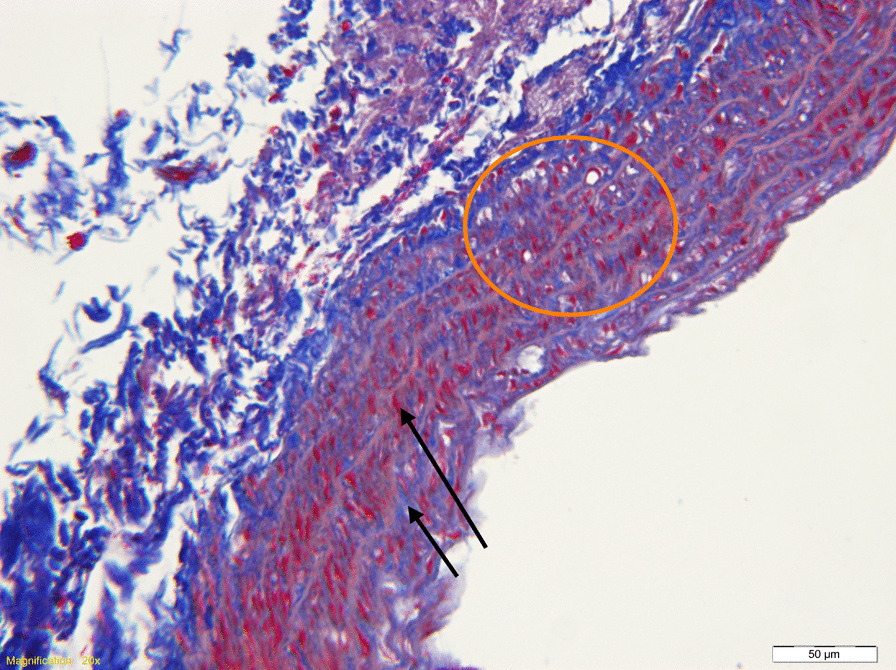
Interpretation of histopathological images of Masson staining (the long black arrow shows the muscle fibers, the short black arrow shows collagen fibers, and orange circles indicate lipid vacuoles)

After the elasticity of the rat abdominal aorta was measured by ultrasound equipment, the animals were euthanized using barbiturate injections before dissection. The bifurcation of the abdominal aorta was searched between the kidneys, and the aorta was separated to the first segment of the aortic arch (Fig. 2). A 1.5–2-cm abdominal aorta was cut 1 cm above the bifurcation of the abdominal aorta, and fixed in 10% paraformaldehyde immediately after the lumen was rinsed with normal saline and phosphate-buffered saline (PBS). After subsequent trimming, dewatering, and paraffin embedding, the 4-μm-thick sections were subjected to Masson staining. The steps were as follows: ① Dewaxing of paraffin sections to water: successively immerse the sections into xylene I 20 min-xylene 20 min-anhydrous ethanol and 10 min-anhydrous ethanol 10 min-95% alcohol 5 min-90% alcohol 5 min-80% alcohol 5 min-70% alcohol 5 min-distilled water for washing. ② Hematoxylin staining for 5 min in the Masson staining kit using Weigert’s ferric hematoxylin, washing with tap water, differentiation with 1% hydrochloric acid and alcohol for a few seconds, and washing with running water for a few minutes to turn blue. ③ Lichun red staining: Lichun red acid fuchsin solution was used in the Masson staining kit for 5–10 min and rinsed with distilled water. ④ Phosphomolybdic acid treatment: The sections were treated with phosphomolybdic acid solution in Masson staining kit for 3–5 min. ⑤ Aniline blue staining: Without washing, the Masson staining kit was directly used for redyeing with aniline blue solution for 5 min. ⑥ Differentiation: 1% acetic acid treatment for 1 min. ⑦ Dehydration sealing: The sections were successively immersed in 95% alcohol I 5 min-95% alcohol II 5 min-anhydrous ethanol I 5 min-anhydrous ethanol 5 min-xylene and 5 min-xylene for 5 min in dehydration and transparency. The sections are taken out of xylene and dried slightly, followed by sealing with neutral gum. Masson stain is specific for compound staining. It can specifically display collagen fibers (blue) and muscle fibers (red). The pathological results showed that the collagen fibrous hyperplasia or the formation of lipid vacuoles is associated with the early arterial wall lesions (Fig. 3).

### Statistical analysis

IBM SPSS 26.0 software was used for statistical analysis. Univariate comparison between the groups was performed using the nonparametric Mann–Whitney U-test, and the diagnostic value was analyzed by the receiver operating characteristic (ROC) curve. P < 0.05 for the area under the curve (AUC) was statistically significant.

## Results

### Histopathological findings of SHR rats

**Fig. 4:**
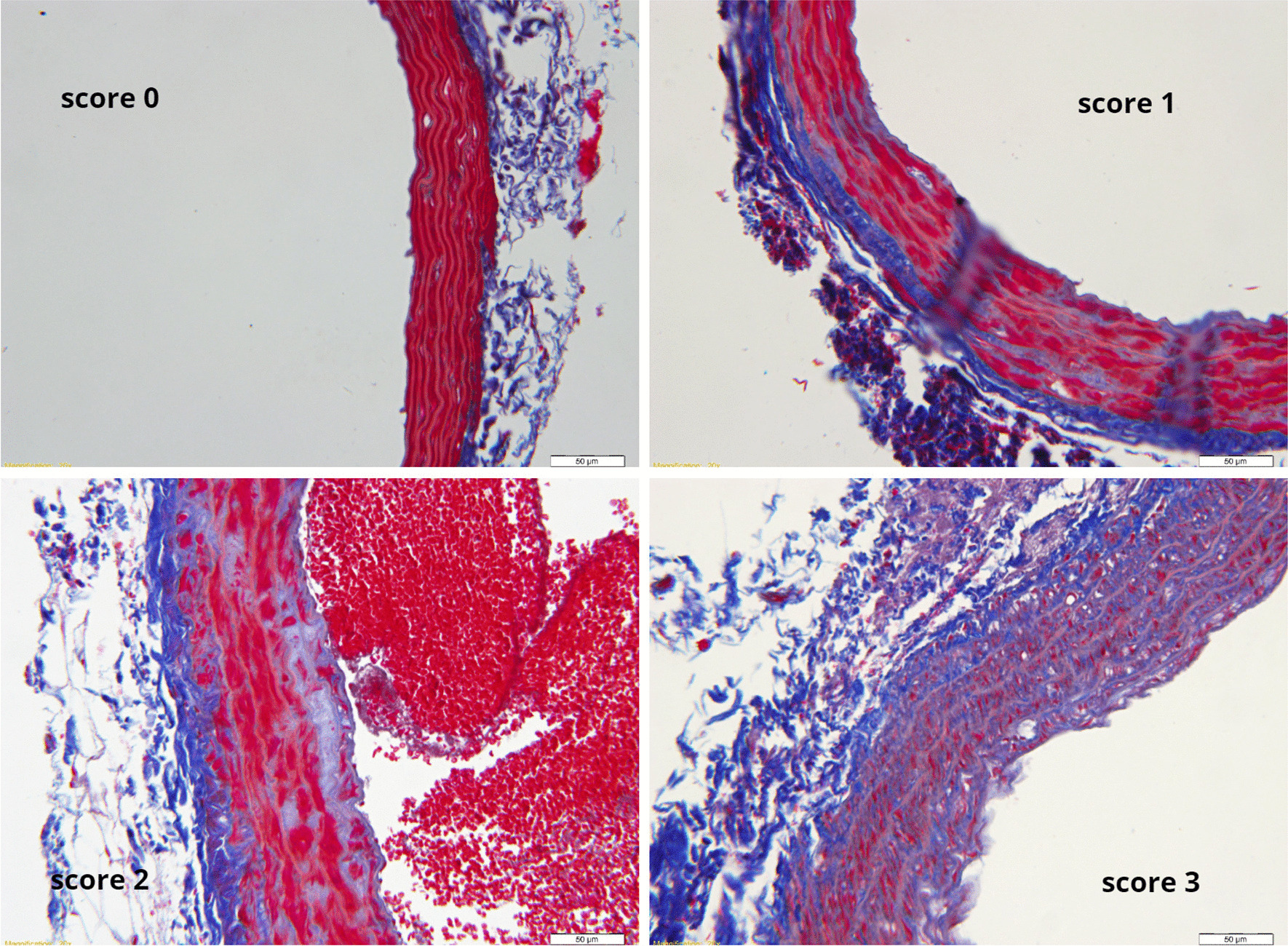
Histopathological findings of SHR rats abdominal aorta (Pathological score 0 is nomal, no collagen fibrous hyperplasia and endodermal lipid vacuoles;score 1 is mild collagen fibrous hyperplasia and no lipid vacuoles; score 2 is moderate and severe collagen fibrous hyperplasia and no lipid vacuoles; score 3 is collagen fibrous hyperplasia and lipid vacuoles)

Since the ultrasound imaging of the abdominal aorta was unsatisfactory and the parameters of elastography deviated greatly, 22 slices from SHR rats were subjected to Masson staining. The final lesion degree was indicated by the total score; the score of no collagen fibrous hyperplasia and lipid vacuoles was 0, the score of mild collagen fibrous hyperplasia and no lipid vacuoles was 1, the score of moderate and severe collagen fibrous hyperplasia and no lipid vacuoles was 2, and the score of collagen fibrous hyperplasia and lipid vacuoles was 3 (Fig. 4).

### Mann–Whitney U-test results

**Fig. 5:**
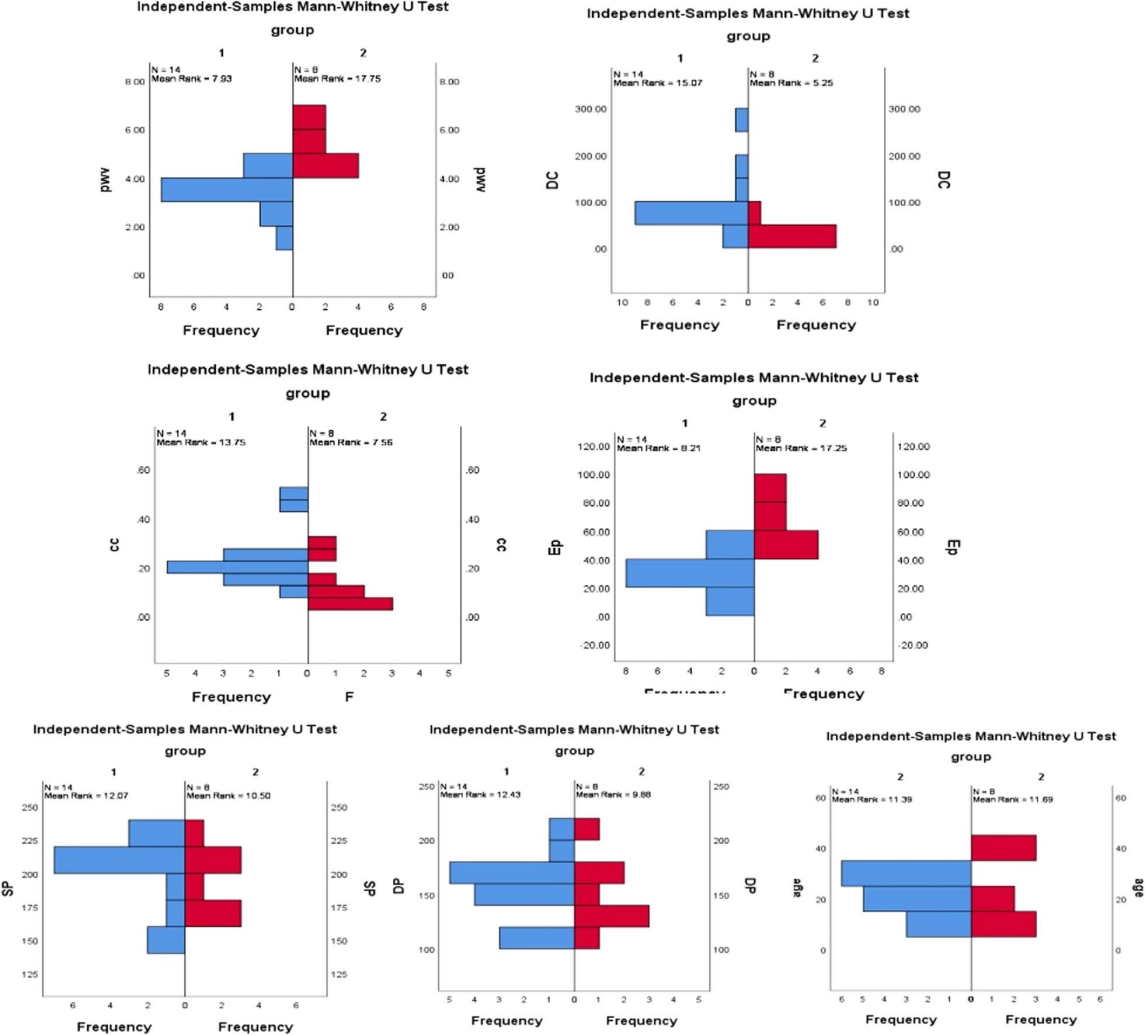
In the Mann–Whitney U-test, frequency distribution of each factor between the two groups (The frequency distributions of PWV, CC, DC, and EP are obviously different; The frequency distributions of SP, DP, Age have no significant differences)

According to the pathological results, the score 0 was divided into the normal group, and the score > 1 was divided into the early arterial wall lesions group. The two groups were compared in terms of age in weeks, blood pressure, and ultrasonic elasticity indices. The results showed significant differences in PWV, CC, DC, and EP (Table 1, Fig. 5).

**Table1:** Mann–Whitney U-test compared the differences between the two groups

Group	Age	PWV	CC	DC	EP	SP	DP
Normal	22.14 ± 8.02	3.57 ± 0.80	0.23 ± 0.11	91.64 ± 61.82	31.91 ± 13.97	199.43 ± 28.33	156.14 ± 29.75
Early arterial wall lesions group	23.75 ± 14.08	5.34 ± 0.97	0.13 ± 0.09	36.04 ± 11.80	64.55 ± 23.22	194.38 ± 23.71	148.25 ± 30.12
P value	0.92	0.001*	0.03*	0.001*	0.002*	0.62	0.40

*Indicated a statistically significant difference

Age(weeks): age of spontaneously hypertensive rats, PWV(m/s): pulse wave velocity, CC (mm^2^/pa): compliance coefficient, DC(1/mpa): distensibility coefficient, EP(Kpa): elasticity parameter, SP (mmHg): Systolic blood pressure, DP (mmHg): Diastolic blood pressure, P < 0.05 indicated a statistically significant difference

### ROC curve analysis results

**Fig. 6:**
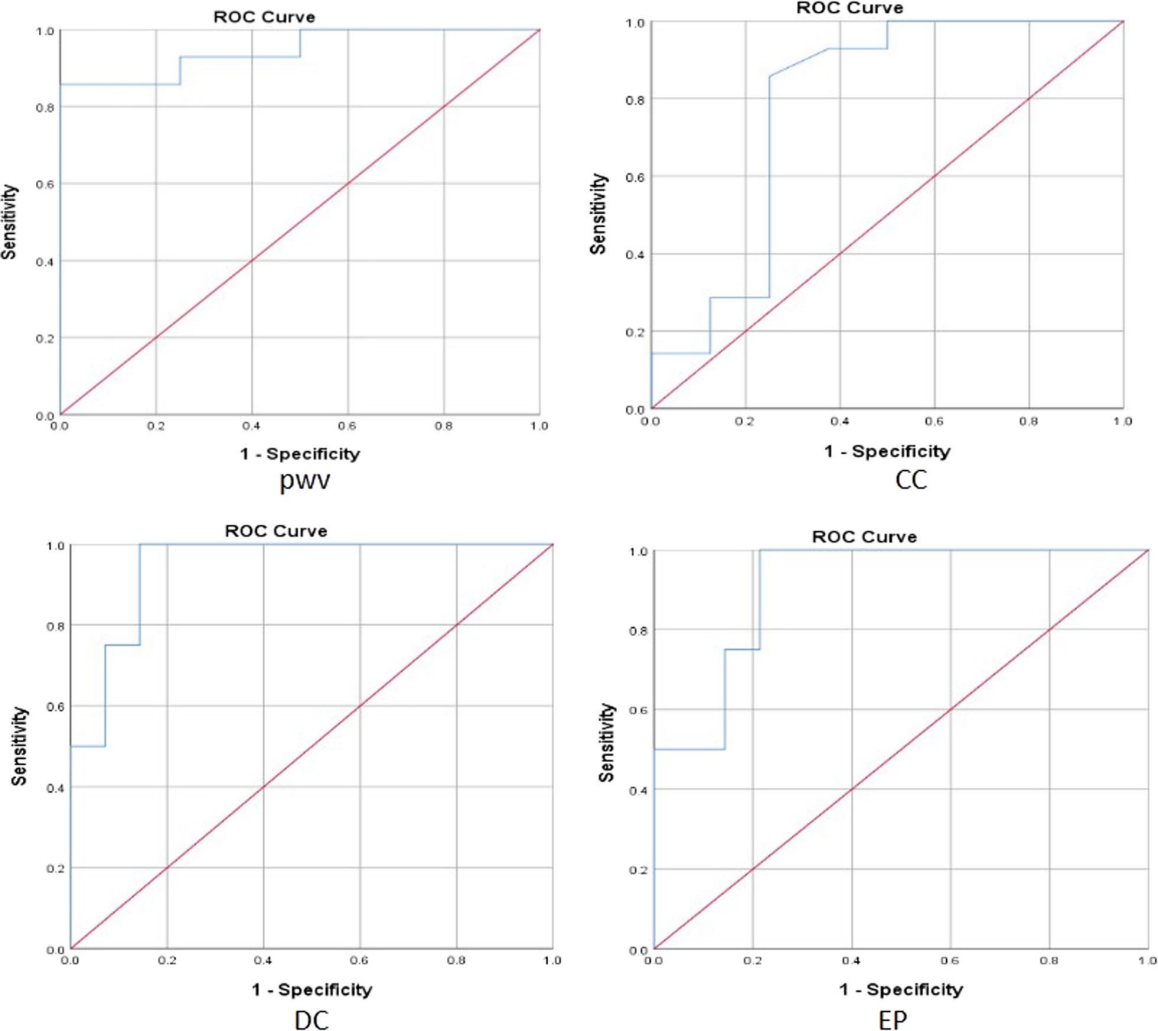
ROC curve of PWV, CC, DC, and EP in predicting the early arterial lesions of SHR (The AUCs of PWV, CC, DC, and EP were 0.946, 0.781, 0.946, and 0.911. The cutoff values for the PWV, CC, DC, and EP were 4.25 m/s, 0.15mm^2^/pa, 52.70 1/mpa, and 39.82Kpa, respectively, with sensitivity and specifificity of 100% and 85.7%, 85.7% and 75%, 85.7% and 100%, and100% and 78.6%).

According to the Mann–Whitney U-test results of the above two groups of rats, the ROC curves were drawn to evaluate the accuracy of PWV, CC, DC, and EP in predicting the early arterial wall lesions of SHR. The level of diagnostic value was indicated by the AUC. In this study, the AUC of PWV for diagnosis of early arteriosclerosis was 0.946 (95% confidence interval (CI) 0.857–1.000, P < 0.05), the AUC of CC was 0.781 (95% CI 0.539–1.000, P < 0.05), the AUC of DC was 0.946 (95% CI 0.857–1.000, P < 0.05), the AUC of EP was 0.911 (95% CI 0.791–1.000, P < 0.05). Also, the cutoff values of each parameter were calculated by Youden index. (Table 2, Fig. 6).

**Table 2:** Sensitivity and specificity of each elastic parameter to predict the early arterial lesions of SHR

Variable	Area under the curve (AUC)	Sensitivity (%)	Specificity (%)	Youden Index	Cutoff value
PWV	0.946	100	85.7	0.857	4.25
CC	0.781	85.7	75	0.607	0.15
DC	0.946	85.7	100	0.857	52.70
EP	0.911	100	78.6	0.786	39.82

*PWV* pulse wave velocity, *CC* compliance coefficient, *DC* distensibility coefficient, *EP* elasticity parameter

## Discussion

In this study, local PWV was measured using ultrasound equipment to assess arterial elasticity. Invasive and non-invasive methods are applied to assess local PWV. The non-invasive evaluation methods include ultrasound and magnetic resonance imaging (MRI) [11.12]. The two main methods for ultrasound assessment of local PWV reported in the literature are vascular echo tracking (ET) [15] and PWV detection based on ultra-high speed imaging technology [14.22]. However, the reports on the above two methods are based on clinical studies, this study is an animal study, the changes of arterial wall elasticity were referenced by histopathology, the results proving the correlation between vascular elastic parameters and arterial wall stiffness are convincing and intuitive.

In addition, multiple vascular elastic parameters(PWV, CC, DC, and EP) were obtained simultaneously in this study. This is different from the above two ultrasonic devices.The parameters obtained by ultra-high speed ultrasound are the pwv at the beginning and at the end of systole (PWV-BS and PWV-ES), and the parameters obtained by ET are arterial compliance (AC), the arterial stiffness index (β), and the single-point pulsed wave velocity (PWVβ) [14, 15, 22]. Meanwhile, the value of the parameters, PWV, CC, DC, and EP, in predicting early arterial lesions by histopathological results was evaluated by ROC curves; the results showed that PWV and DC had high diagnostic values. The AUC was 0.946, the PWV diagnostic sensitivity was 100%, and the specificity was 85.7%. Moreover, the DC diagnostic sensitivity was 85.7%, and the specificity was 100%, which was consistent with a previous report. PWV is the gold standard for evaluating arterial stifness, and arterial stiffness has independent predictive value for cardiovascular events in hypertensive patients [23]. DC is a critical parameter for predicting cardiovascular diseases, a decrease in DC of 1 SD predicted a 13%, 6%, and 41% higher incidence of cardiovascular events,cardiovascular mortality and all-cause mortality, respectively, whereas a decrease in DC of 1 unit predicted a 3%, 1% and 6% higher incidence of the corresponding clinical events [24]. Therefore, the method applied in this study is feasible for the assessment of early arterial wall lesions.

Many clinical factors, such as age, blood pressure, heart rate, and other cardiovascular risk factors, can affect the measured values of vascular elasticity parameters [25]. Among these, the most robust confounding factor is blood pressure, which increases the tension of arterial wall and the degree of arterial stiffness [26]. Therefore, the influence of blood pressure when measuring vascular elasticity parameters needs to be investigated further. In this study, no statistically significant difference was observed in the blood pressure between the normal and the early arteriosclerosis group, which might be caused by the small sample size, or the blood pressure was not monitored in real time, or the influence of arterial stiffness on vascular elasticity parameters was greater than that of blood pressure. Next, we will measure the vascular elasticity parameters under real-time blood pressure monitoring in the population to evaluate the correlation between blood pressure and the measured values of vascular elasticity parameters.

Furthermore, no significant difference was observed in age between the normal and the early arteriosclerosis groups. This phenomenon was different from that reported previously. Some studies have shown that PWV increases with age as arterial stiffness increases in humans [27], which could be attributed to the small difference in the number of weeks of rats selected in this study, but did not cause obvious differences in arterial wall elasticity. Together, these findings indicated elastography parameters can accurately evaluate the stiffness of arterial wall.

## Conclusions

A new ultrasound device was used to evaluate the elastic function of the abdominal aorta in rats in this study. Four elastic parameters were obtained, including PWV, CC, DC, and EP. Significant differences were observed in the above four elastic parameters between the normal and the early arterial wall lesions groups. The ROC curve analysis showed that the sensitivity and specificity of PWV and DC were high, which could be used in combination to evaluate the arterial elasticity. Also, no statistically significant difference was detected in blood pressure and weeks of age between the normal and the early arterial wall lesions groups, which is different from previous studies, indicating that elastography parameters can accurately assess arterial elastic function without being affected by other factors.

## Data Availability

Since the follow-up experiments are ongoing, the raw data cannot be shared here. However, the datasets used and/or analyzed during the current study are available from the corresponding author upon reasonable request.

## Abbreviations

AUC: Area under the curve
CC: Compliance coefficient
CI: Confidence interval
DC: Distensibility coefficient
DP: Diastolic blood pressure
EP: Elasticity parameter
PBS: Phosphate-buffered saline
PP: Pulse pressure
PWV: Pulse wave velocity
ROC: Receiver operating characteristic curve
SHR: Spontaneous hypertensive rats
SP: Systolic blood pressure
SPF: Specific pathogen-free

## Declarations

### Declarations

Authors confirm that all methods are reported in accordance with ARRIVE guidelines (https://arriveguidelines.org) for the reporting of animal experiments.
